# Azithromycin and Roxithromycin define a new family of “senolytic” drugs that target senescent human fibroblasts

**DOI:** 10.18632/aging.101633

**Published:** 2018-11-14

**Authors:** Bela Ozsvari, John R. Nuttall, Federica Sotgia, Michael P. Lisanti

**Affiliations:** 1 Translational Medicine, University of Salford, Greater Manchester, United Kingdom; * Equal contribution

**Keywords:** drug repurposing, aging, senescence, senolytic drugs, antibiotics, azithromycin, roxithromycin

## Abstract

Here, we employed a “senolytic” assay system as a screening tool, with the goal of identifying and repurposing FDA-approved antibiotics, for the targeting of the senescent cell population. Briefly, we used two established human fibroblast cell lines (MRC-5 and/or BJ) as model systems to induce senescence, via chronic treatment with a DNA-damaging agent, namely BrdU (at a concentration of 100 μM for 8 days). Cell viability was then monitored by using the SRB assay, to measure protein content. As a consequence of this streamlined screening strategy, we identified Azithromycin and Roxithromycin as two novel clinically-approved senolytic drugs. However, Erythromycin – the very closely-related parent compound – did not show any senolytic activity, highlighting the dramatic specificity of these interactions. Interestingly, we also show that Azithromycin treatment of human fibroblasts was indeed sufficient to strongly induce both aerobic glycolysis and autophagy. However, the effects of Azithromycin on mitochondrial oxygen consumption rates (OCR) were bi-phasic, showing inhibitory activity at 50 μM and stimulatory activity at 100 μM. These autophagic/metabolic changes induced by Azithromycin could mechanistically explain its senolytic activity. We also independently validated our findings using the xCELLigence real-time assay system, which measures electrical impedance. Using this approach, we see that Azithromycin preferentially targets senescent cells, removing approximately 97% of them with great efficiency. This represents a near 25-fold reduction in senescent cells. Finally, we also discuss our current results in the context of previous clinical findings that specifically document the anti-inflammatory activity of Azithromycin in patients with cystic fibrosis – a genetic lung disorder that results in protein mis-folding mutations that cause protein aggregation.

## Introduction

As a diversity of organism(s) undergo chronological aging, many genetic, phenotypic and metabolic defects accumulate, including the onset of senescence in a variety of cell types [1]. This overall view is consistent with the “accumulated damage” hypothesis of aging [2,3].

Senescence is a clear hallmark of normal chronological aging. Senescence involves potentially irreversible cell cycle arrest, via the induction of CDK-inhibitors, such as p16-INK4A, p19-ARF, p21-WAF and p27-KIP1, as well as the onset of the SASP (senescence-associated secretory phenotype) [4], and the induction of key lysosomal enzymes (e.g., Beta-Galactosidase) and Lipofuscin, an established aging-pigment [5]. Interestingly, SASP results in the secretion of a wide array of inflammatory cytokines, such as IL-1-beta and IL-6, allowing senescent cells to “contagiously” spread the senescence phenotype from one cell type to another, systemically throughout the body, via chronic inflammation. Such chronic inflammation can also promote the onset of cancer, as well as drive tumor recurrence and metastasis [6, 7].

Using the promoter of p16-IN4KA as a transgenic probe to detect and mark senescent cells, several research groups have now created murine models of aging in which senescent cells can be genetically eliminated in a real-time temporal fashion [8,9]. Although this cannot be used as an anti-aging therapy, it can give us an indication whether the removal of senescent cells can potentially have therapeutic benefits to the organism. Results to date show great promise, indicating that the genetic removal of senescent cells can indeed prolong healthspan and lifespan [10,11].

As a consequence of this exciting genetic data, a large number of pharmaceutical companies are now actively engaged in the discovery of “senolytic” drugs that can target senescent cells. However, we believe that many FDA-approved drugs may also possess senolytic activity and this would dramatically accelerate the clinical use of these senolytic drugs in any anti-aging drug trials. 

Here, we have used controlled DNA-damage as a tool to induce senescence in human fibroblasts, which then can be employed as an efficient platform for drug screening. More specifically, we employed BrdU-treatment, which has a long history of being used as a DNA-damaging agent, to reproducibly induce senescence in cultured cells, with high efficiency [12-17]. 

Using this approach, we now report the identification of two macrolide antibiotics of the Erythromycin family, specifically Azithromycin and Roxithromycin, as new clinically-approved senolytic drugs. In direct support of the high specificity of these complex interactions, the parent macrolide compound – Erythromycin itself – has no senolytic activity in our assay system. 

## RESULTS

### Detection and characterization of “senolytic” activity during the screening of clinically-approved therapeutics

**Figure 1:**
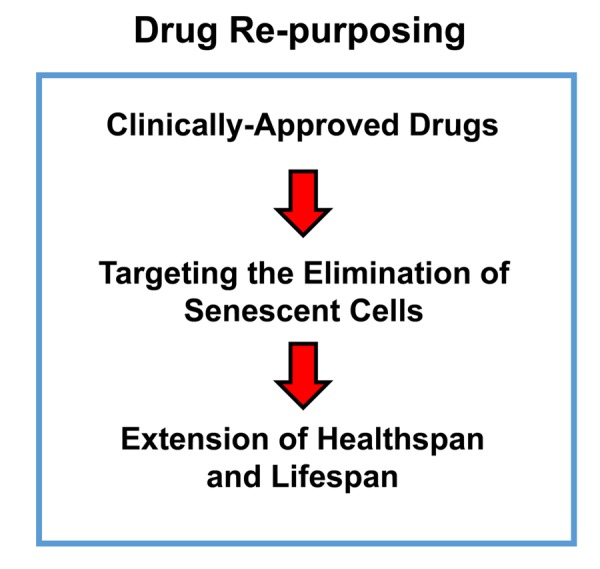
**Targeting senescent cells with clinically-approved drugs.** Here, we propose to use clinically-approved drugs, including antibiotics, to target and eliminate senescent cells, with the goal of increasing healthspan and lifespan.

Here, we used a simplified screening assay to identify and repurpose clinically-approved therapeutics with “senolytic” activity for the treatment of aging and aging-associated disorders (Figure 1).

**Figure 2:**
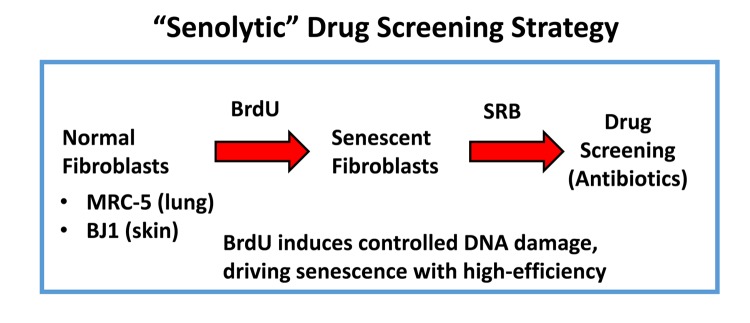
**“Senolytic” drug screening strategy.** Here, normal fibroblasts (MRC-5 and BJ), originally derived from human lung and skin tissues, were subjected to prolonged culture (8-days) in the presence of BrdU (100 μM) to induce controlled DNA-damage and senescence. Then, isogenically-matched cultures of normal and senescent fibroblasts were employed for drug screening to identify the potential senolytic activity of clinically-approved drugs, such as antibiotics (Erythromycin, Azithromycin and Roxithromycin, among others). Senolytic activity was detected using the SRB assay, which measures the amount of protein remaining attached to the tissue-culture dishes, which is a surrogate marker for cell viability.

More specifically, we employed two independent normal, non-immortalized, human fibroblast cell lines, namely i) MCR-5 for screening and ii) BJ for validation (Figure 2). Mechanistically, the responses of “normal” fibroblasts and “senescent” fibroblasts were directly compared, side-by-side. Drugs that preferentially killed senescent fibroblasts, but not normal fibroblasts, were considered as a positive hit. Using this approach, we identified two Erythromycin-family members, Azithromycin and Roxithromycin that preferentially targeted senescent fibroblasts (Table 1). However, Erythromycin itself did not show any senolytic activity.

**Figure:**
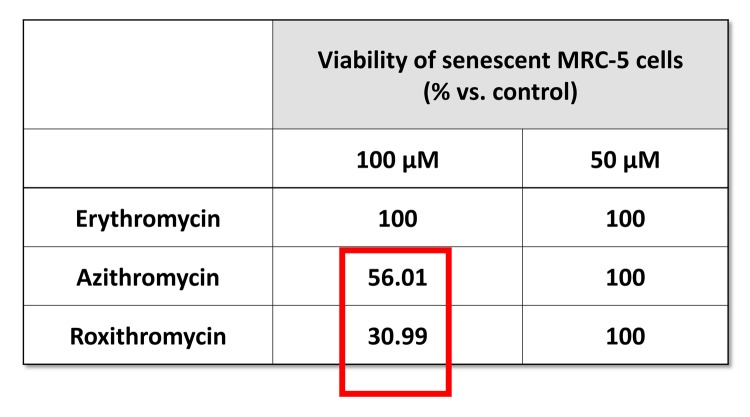
**Table 1. The effects of macrolide antibiotics on BrdU-treated senescent MRC-5 fibroblasts.** This table briefly summarizes the biological effects of three antibiotics, namely Erythromycin, Azithromycin and Roxithromycin, on cell viability. While Erythromycin was completely ineffective, Roxithromycin and Azithromycin selectively eliminated large numbers of senescent cells at 100 µM, but had no effect at a lower dose (50 µM). Azithromycin was found to be the most selective compound, as it eliminated senescent cells, without affecting control cells.

**Figure 3:**
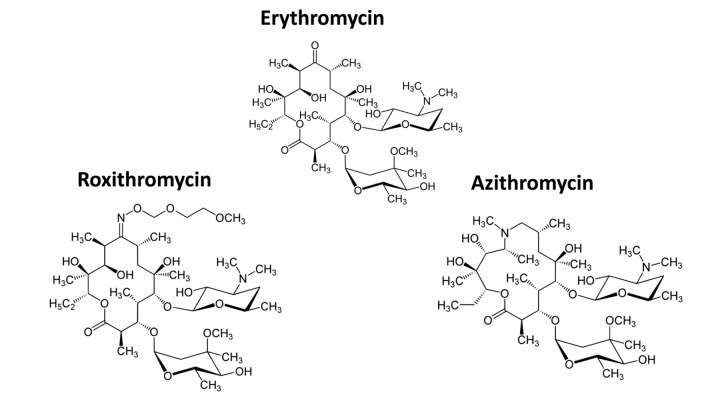
**Chemical structures of Erythromycin and related compounds.** Note that Erythromycin, Azithromycin and Roxithromycin all have very similar chemical structures, but differ mainly in their side groups. All three compounds are macrolide antibiotics and consist of a large core macrocyclic lactone ring, with two deoxy-sugars attached to it.

Figure 3 shows a comparison of the precise chemical structures of the Erythromycin family members we tested. Note that the compounds are nearly identical, suggesting highly specific interactions must underpin the senolytic activity of Azithromycin and Roxithromycin.

**Figure 4:**
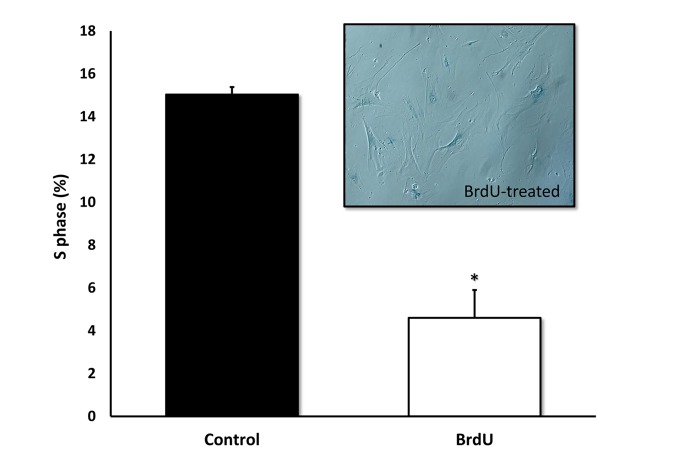
**BrdU-treatment of MRC-5 cells effectively inhibits DNA-synthesis and induces Beta-Galactosidase.** Two-day treatment with BrdU significantly reduced DNA synthesis in MRC-5 fibroblasts by ~70%, as measured with the Muse cell cycle kit. MRC-5 cells after 8 days of BrdU treatment were positively stained for Beta-Galactosidase. n=3; * p < 0.05.

Furthermore, we validated previous findings that BrdU-induced DNA-damage is indeed sufficient to induce cellular senescence. Figure 4 shows that MRC-5 fibroblasts treated with BrdU underwent cell cycle arrest, as evidenced by i) a ~70% reduction in the number of cells in S-phase and ii) the induction of Beta-Galactosidase activity.

**Figure 5:**
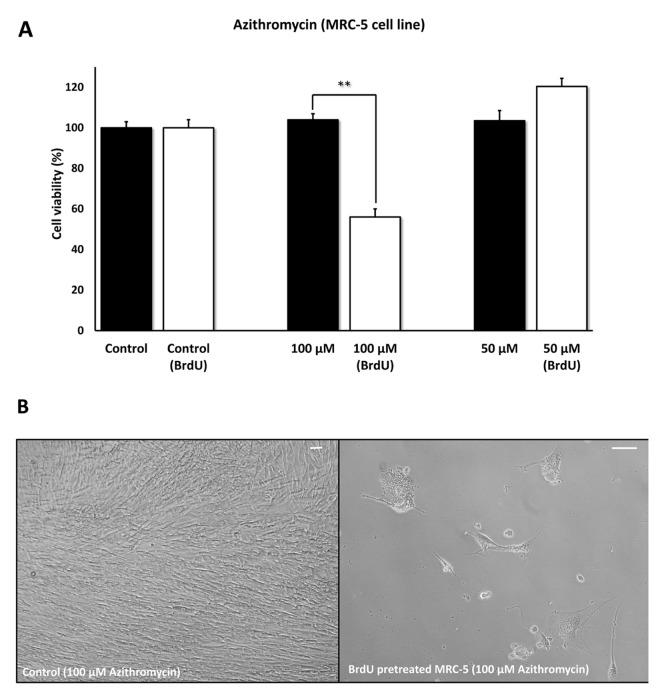
**Azithromycin shows senolytic activity in senescent MRC-5 human lung fibroblasts.** MRC-5 cells were pre-treated with BrdU for 8 days (to induce senescence), before they were exposed to Azithromycin for another 5 days. After that, the SRB assay was performed to determine the effects of the drug on cell viability. Azithromyin had a potent and selective effect on MRC-5, as it eliminated ~50% of senescent cells without affecting control cells after 5 days, at a concentration of 100 µM. However, Azithromycin had no effect at 50 µM. These experiments were repeated at least 3 times independently, with very similar results. Note that the scale bar represents 20 µm in the images. ** p < 0.01.

**Figure 6:**
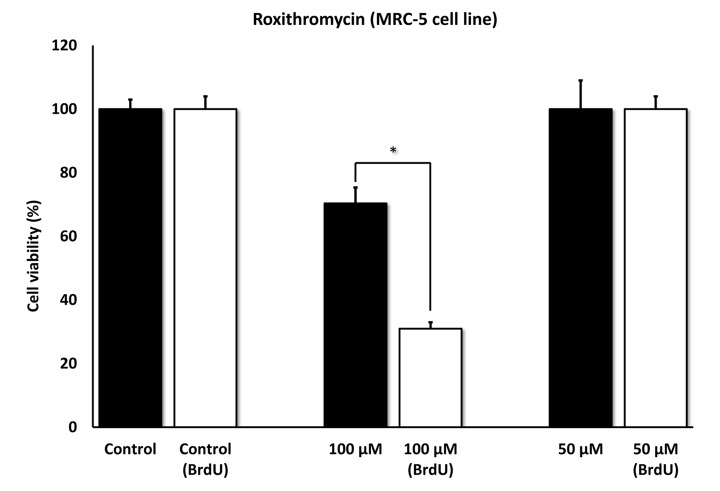
**Roxithromycin shows senolytic activity in senescent MRC-5 human lung fibroblasts.** MRC-5 cells were pre-treated with BrdU for 8 days (to induce senescence), before they were exposed to Roxithromycin for another 5 days. After that, the SRB assay was performed to determine the effects of the drug on cell viability. Roxithromycin had a potent and selective effect on MRC-5, as it eliminated more than 50% of senescent cells after 5 days, at a concentration of 100 µM. However, Roxithromycin had no effect at 50 µM. These experiments were repeated at least 3 times independently, with very similar results. * p < 0.05.

Figures 5 directly shows that Azithromycin, at 100 μM, had no effect on the viability of normal MRC-5 lung fibroblasts, but selectively killed only senescent MCR-5 fibroblasts. In comparison, Roxithromycin, at the same concentration, more effectively killed senescent MCR-5 fibroblasts (~70%), but also had a small effect on the viability of normal MRC-5 fibroblasts (Figure 6). Neither drug showed any significant effects on viability at 50 μM, indicating that the effects we observed were concentration-dependent. As such, Azithromycin toxicity showed the highest specificity for selectively targeting the senescent cell phenotype.

### Phenotypic and metabolic effects of Azithromycin in MRC-5 human fibroblasts

To better mechanistically understand the phenotypic and metabolic effects of Azithromycin, we employed normal MRC-5 fibroblasts.

**Figure 7:**
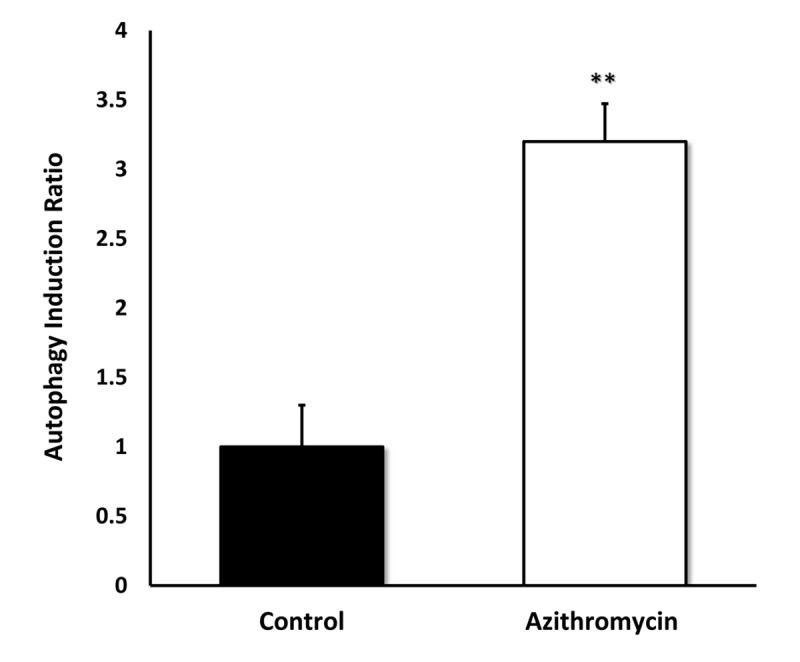
**Azithromycin strongly induces autophagy in MRC-5 cells.** MRC-5 cells were treated with Azithromycin at 50 µM for 72 hours. Then, autophagy was monitored by detection of autophagic LC3 proteins with the Muse Autophagy LC3-antibody based kit. Azithromycin treatment resulted in more than a 3-fold elevation in autophagy in MRC-5 cells.

Figure 7 shows that Azithromycin is a powerful inducer of the autophagic phenotype. Autophagy was quantitatively measured by detection of autophagic LC3 proteins, using the Muse Autophagy LC3-antibody based kit. Note that Azithromycin treatment resulted in a > 3-fold elevation in autophagy in MRC-5 cells.

**Figure 8:**
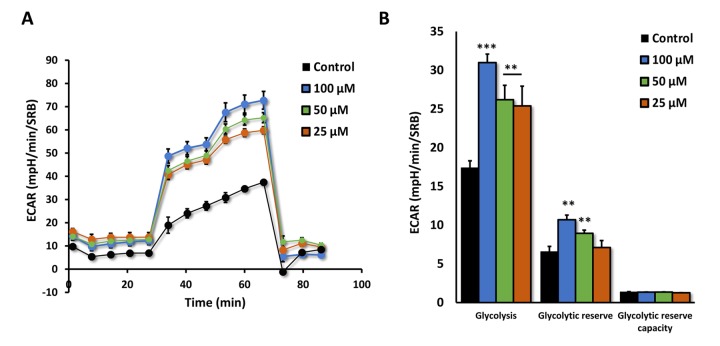
**Azithromycin induces glycolytic activity in MRC-5 cells.** After 72 hours of treatment with Azithromycin (25 to 100 µM), MRC-5 cells were subjected to metabolic flux analysis with the Seahorse XFe96, which measures ECAR (extracellular acidification rate). Note that all concentrations elevated glycolysis. n=3; ** p < 0.01,*** p < 0.001.

We next measured the effects of Azithromycin on i) aerobic glycolysis and ii) mitochondrial metabolism, using the Seahorse XFe96 metabolic flux analyzer. Figure 8 shows that even low concentrations of Azithromycin (25 μM) effectively induced glycolytic flux in MRC-5 fibroblasts, in the presence of oxygen.

**Figure 9:**
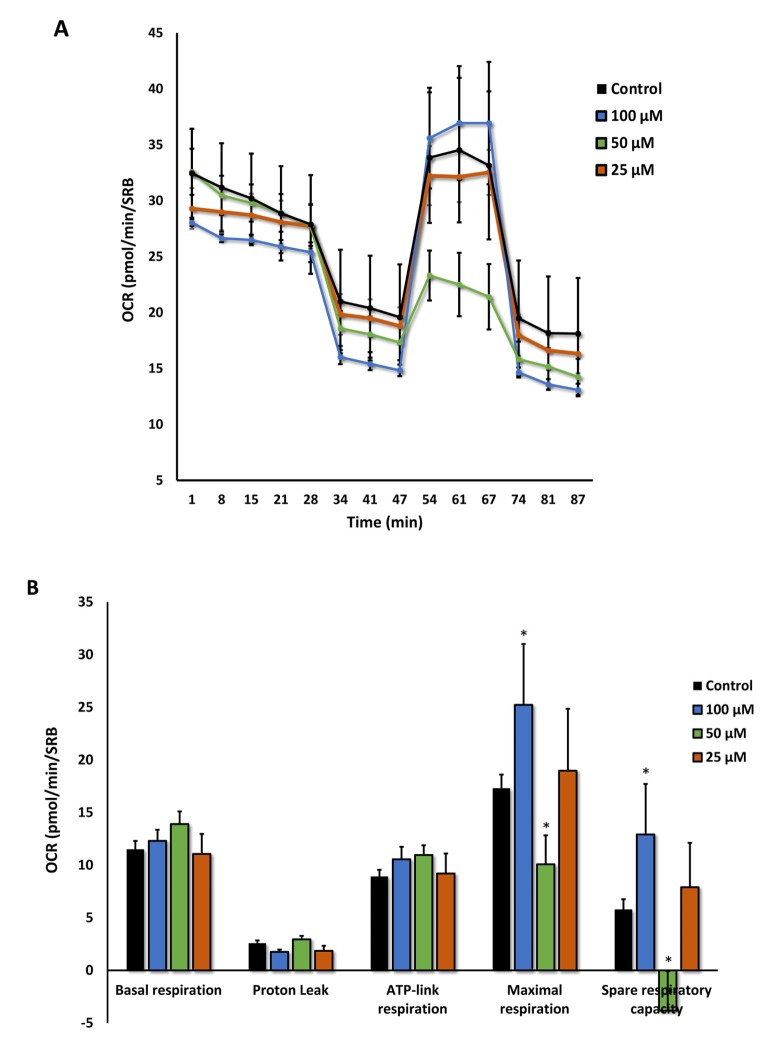
**Azithromycin has biphasic effects on oxygen consumption in MRC-5 cells.** After 72 hours of treatment with Azithromycin (25 to 100 µM), MRC-5 cells were subjected to metabolic flux analysis with the Seahorse XFe96, which measures OCR (the oxygen consumption rate). Note that the highest dose (100 µM) triggered increased mitochondrial respiration, while the lower concentrations (50 µM) significantly reduced it. However, 25 µM did not have any significant effects on OCR. n=3; * p < 0.05.

Moreover, the effects of Azithromycin on mitochondrial oxygen consumption rate (OCR) are highlighted in Figure 9A,B. Note that the mitochondrial effects of Azithromycin were concentration-dependent and bi-phasic. At 25 μM, Azithromycin did not show any effects on OCR. However, at 50 μM, the effects of Azithromycin clearly inhibited mitochondrial metabolism, especially effecting maximal respiration and spare respiratory capacity. In contrast, at 100 μM, Azithromycin actually stimulated maximal respiration and more than doubled spare respiratory capacity. This may represent a cellular compensatory response to Azithromycin treatment, to overcome its mitochondrial inhibitory effects.

### Validating the selectivity and potency of Azithromycin using BJ human fibroblasts

**Figure 10:**
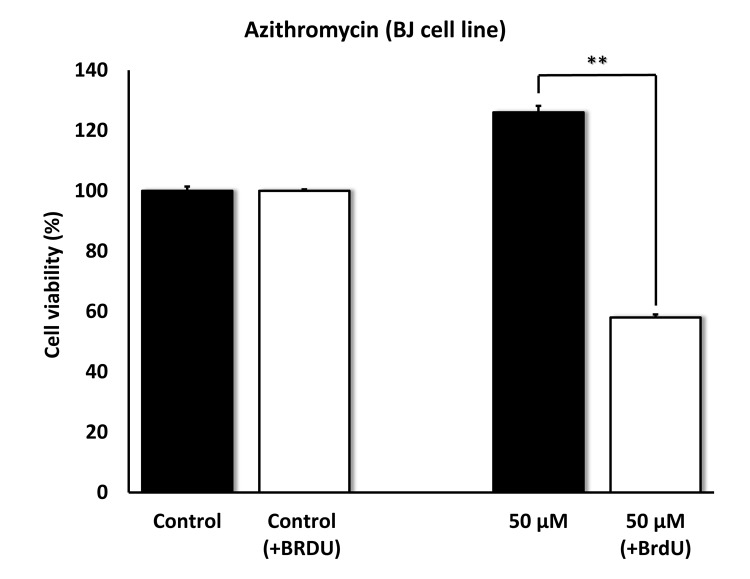
**Azithromycin also shows senolytic activity in senescent BJ human skin fibroblasts.** BJ cells were pretreated with BrdU for 8 days (to induce senescence), before they were exposed to Azithromycin for another 5 days. After that, SRB assay was performed to determine the effects of Azithromycin on cell viability. Azithromyin had a potent and selective effect on BJ cells, as it eliminated > 50% of senescent cells without reducing the viability of control cells after 5 days at 50 µM. These experiments were repeated at least 3 times independently, with very similar results. ** p<0.01.

To further validate the senolytic activity of Azithromycin, we also assessed its selective effects by employing normal, non-immortalized, BJ human skin fibroblasts. Figure 10 shows that Azithromycin was more potent in BJ skin fibroblasts, showing significant “senolytic” activity at only 50 μM. Remarkably, Azithromycin also increased the viability of normal BJ skin fibroblasts, by > 25%. As such, Azithromycin shows comparable selectively and senolytic activity in human fibroblasts derived from two different anatomic sites (lung tissue and skin).

### Other drug candidates tested do not show any senolytic activity

In parallel, we also tested a number of other drug candidates, using this senolytic assay system employing MRC-5 or BJ fibroblasts. Unfortunately, none of these other drug candidates showed any specific senolytic activity, while sparing their normal fibroblast counterparts. These findings further highlight the specificity and selectivity of Azithromycin and Roxithromycin in the targeting of senescent cells. These results are included as Supplementary Table S1.

### Independent validation of the high selectivity of Azithromycin for targeting senescent cells using the xCELLigence system

Because senescent cells undergo the so-called senescence-associated secretory phenotype (SASP), which involves dramatic increases in the synthesis and secretion of proteins [1–7], we were concerned that our assay system – which measures protein – might be actually under-estimating the ability of Azithromycin to target senescent cells.

**Figure 11:**
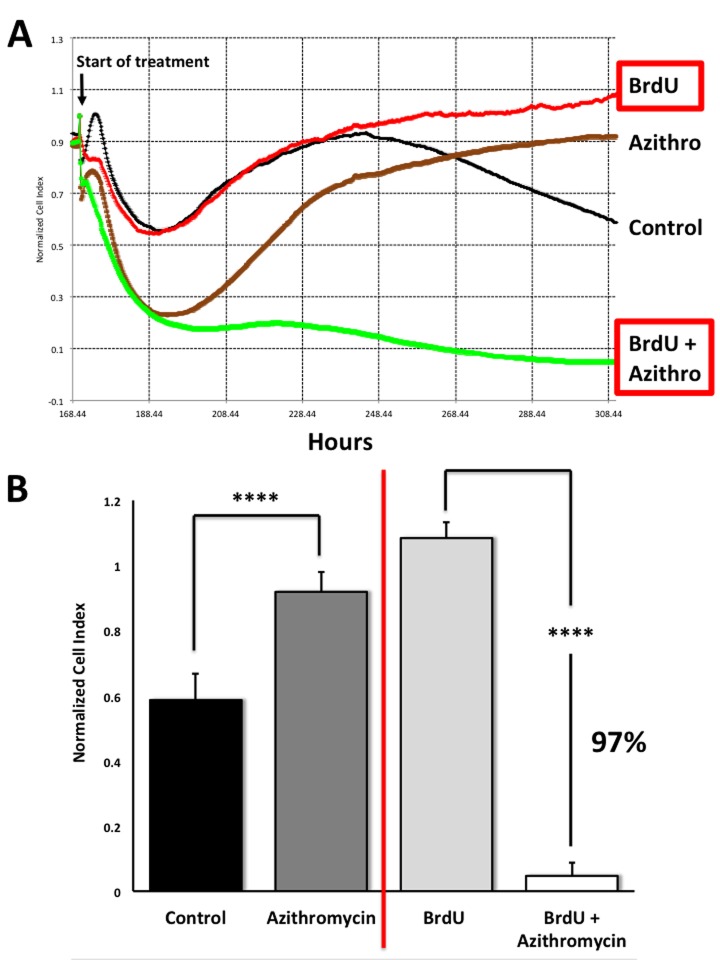
**Assessing the efficacy of Azithromycin in the selective targeting of senescent MRC-5 cells, using the xCELLigence system.** The xCELLigence system allows for the real-time, label-free, monitoring of cell health and behavior, via high frequency measurement of cell-induced electrical impedance. Panel (**A**) A representative cell tracing is shown. Note that senescent cells (BrdU-treated/MRC-5 fibroblasts) were effectively killed; directly compare the red curve (BrdU only) versus the green curve (BrdU plus Azithromycin). For normal MRC-5 fibroblasts, compare the black curve (Control) with the brown****curve (Azithromycin). Panel (**B**) Bar graphs highlighting the final cell index, are shown as the average ^+^ the SEM. Approximately 97% of the senescent MRC-5 cells are targeted by Azithromycin treatment. In contrast, normal control MRC-5 cells are only transiently affected by Azithromycin, and they rapidly recover via cell proliferation, exceeding vehicle-alone control cell levels by greater than 30%. **** p < 0.001.

To address this issue directly, we used another independent assay system (called xCELLigence) that does not depend on proteins, but instead uses electrical impedance to continuously measure cell proliferation, in a real-time fashion. Using this approach, we see that Azithromycin preferentially targets senescent cells, removing approximately 97% of them with great efficiency (Figure 11). This represents a near 25-fold reduction in senescent cells (p < 0.001).

This real-time analysis also revealed that the population of normal control MRC-5 cells are also transiently affected by Azithromycin, but that they rapidly recover through additional cell proliferation (Figure 11), exceeding vehicle-alone control cell levels by > 30% (p < 0.001).

Therefore, this real-time assay system is superior to our static SRB assay, for more directly visualizing the potential “senolytic” effects of compounds, during drug screening.

## DISCUSSION

### New senolytic drugs: Azithromycin and Roxithromycin

In this report, we used a “senolytic” screening approach to systematically identify clinically-approved drugs to target the senescence phenotype of human fibroblasts. For this purpose, we employed MCR-5 and BJ cells (two well-established non-immortalized human fibro-blast cell lines), treated with BrdU - a DNA-damaging agent. Briefly, to induce cell cycle arrest and senescence, fibroblasts were exposed to BrdU (100 μM) to an 8-day period. After drug treatment for another 5 days, cell attachment was assessed via the SRB assay system, using a plate-reader, allowing high throughput analysis. As a result, using this screening approach, we discovered that two clinically-approved macrolide antibodies (namely, Azithromycin and Roxithromycin) preferentially exhibited “senolytic activity”. In contrast, the nearly identical drug, Erythromycin, did not show any toxicity towards senescent fibroblasts, directly demonstrating the high-specificity of the actions of Azithromycin and Roxithromycin. Metabolic analysis of the chemical effects of Azithromycin showed that it induced the onset of i) autophagy and ii) glycolysis. Moreover, Azithromycin increased mitochondrial activity at high dose (100 μM), but had the opposite effect at a lower dose (50 μM), demonstrating clear bi-phasic effects. We believe that these metabolic effects of Azithromycin could underpin its highly specific senolytic activity

Interestingly, Azithromycin is used clinically to chronically treat patients with cystic fibrosis [18], a genetic disease of the chloride-transporter, that generates a hyper-inflammatory state in lung tissue [19]. This is due to mutations that result in the mis-folding of the chloride transporter, followed by its degradation by proteases (most commonly the CFTR-ΔF508 mutation) [20]. In this context, Azithromycin extends patient lifespan by acting as an anti-inflammatory drug that prevents the onset of lung fibrosis by targeting and somehow eliminating “pro-inflammatory” lung fibroblasts [21,22]. Therefore, the efficacy of Azithromycin in cystic fibrosis patients provides supporting clinical evidence for our current findings, as these lung fibroblasts are pro-inflammatory most likely because they are senescent. Although this remains to be formally proven, it is well-known that the SASP generate a plethora of pro-inflammatory cytokines that rapidly spread the senescence phenotype to other neighboring cells and tissues, a phenomenon known as “inflamm-aging”. Therefore, it may be useful to think about cystic fibrosis as a pro-aging disease, because of its genetic association with protein mis-folding, resulting in chronic inflammation and thereby driving shortened lifespan.

In accordance with our current findings, Azithromycin treatment is also protective against radiation-induced lung damage in mice. Radiation is considered as a pro-aging stimulus, as it accelerates DNA damage, ROS production and oxidative stress, as well as inflammation and fibrosis. In this model system, Azithromycin treatment morphologically inhibited the onset of lung inflammation and fibrosis, as well as quantitatively prevented the accumulation of markers of i) oxidative stress (MDA; malondialdehyde), ii) inflammation (IL1-beta, IL-6, TNA-alpha) and iii) fibrosis (TGF-beta-1, Alpha-SMA, Collagen I) [23]. In the context of TGF-beta-1, Collagen I and Alpha-SMA, it has also been proposed that Azithromycin prevents the onset of the myo-fibroblast phenotype [24]. However, in light of our current data, we suggest instead that myo-fibroblasts are actually senescent cells, which are rapidly and effectively eliminated by Azithromycin

In addition, Roxithromycin has been shown to effectively promote hair re-growth [25-27], possibly by stimulating the production of normal hair follicle stem cells. Mechanistically, this hair re-growth phenomenon could be due to the highly efficient removal of neighboring senescent skin fibroblasts. Moreover, Roxithromycin has also been reported to have stronger anti-inflammatory effects, than both Azithromycin and Erythromycin [28]. 

### Does autophagy confer “senolytic” activity?

**Figure 12:**
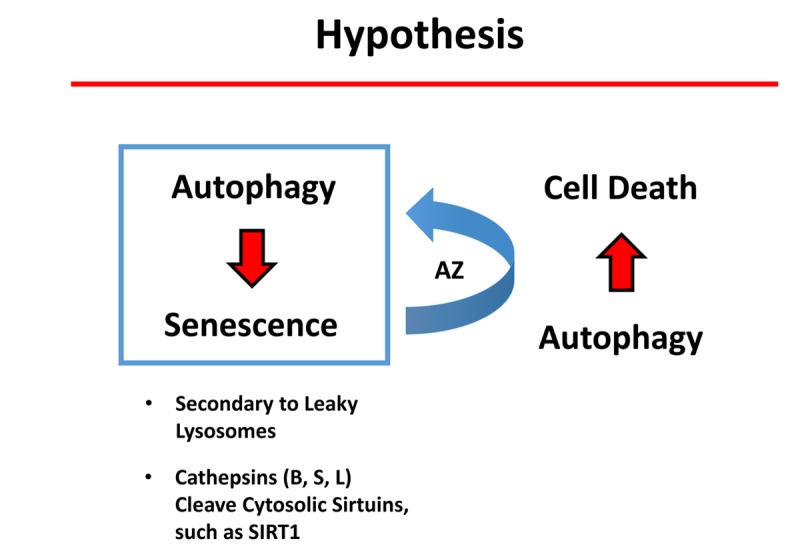
**Potential role of autophagy in conferring “senolytic” activity.** Autophagic cells have an increased tendency to become senescent. Mechanistically, autophagic cells accumulate large numbers of lysosomes and auto-phagosomes. These organelles contain high levels of proteases, such as cathepsins (B, S and L). Interestingly, it has been previously demonstrated that lysosomes in authophagic cells can become “leaky” due to an acute stress, ultimately resulting in stress-induced senescence (SIS). As a consequence, cathepsins leak into the cytoplasm where they can then cleave sirtuin family members (e.g., SIRT1), paving the way for the onset of senescence. Here, we show that a weak autophagy inducer, Azithromycin (AZ), selectively targets senescent cells. We speculate that the weak induction of autophagy in senescent cells can result in cell death.

It is now well-established that autophagic cells have an increased likelihood of becoming senescent. This is called the autophagy-senescence transition (AST). More specifically, during autophagy, cells accumulate auto-phagic organelles (lysosomes and auto-phagosomes). These structures normally sequester dangerous proteases, including the cathepsins (B, S and L). However, during an acute stress, lysosomes in autophagic cells can become “leaky”, resulting in the release of the cathepsins into the cytosol, secondrary to lysosome rupture or defects in the lysosomal membrane [29]. Once in the cytosol, the cathepsins can proteolytically cleave the sirtuins, such as SIRT1, driving the senescence pheno-type [29]. Interestingly, we show here that Azithromycin, a weak autophagy inducer, preferentially targets senescent cells. Thus, we speculate that the induction of autophagy in senescent cells can drive cell death (Figure 12). Undoubtedly, further experimentation will be required to test this attractive new hypothesis directly.

## CONCLUSIONS

In summary, we conclude that it is possible to identify pre-existing clinically-approved antibiotics with senolytic activity, for drug repurposing as anti-aging drugs that can be used to target senescent fibroblasts. The specific examples we provide are Azithromycin and Roxithromycin, two well-known macrolide antibiotics.

## MATERIALS AND METHODS

### Materials

MRC-5 (ATCC^®^ CCL-171) human lung fibroblast cells and BJ (ATCC^®^ CRL2522) human skin fibroblasts were purchased from the ATCC (American Type Culture Collection). Gibco-brand cell culture media (MEM) was purchased from Life Technologies. Bromodeoxyuridine, Azithromycin, Roxithromycin and Erythromycin were purchased from Sigma-Aldrich. Azithromycin (from Pfizer) is FDA-approved. Roxithromycin (from GSK and Sandoz) is not available in the United States, but is clinically-approved in New Zealand, Australia and Israel.

### Experimental design

MRC-5 or BJ cells were plated into 24-well plates. Next day, half of the plate was treated with 100 µM of Bromodeoxyuridine (BrdU) while control wells were treated with vehicle only (DMSO) and incubated for 8 days at 37°C in a 5% CO2 humidified atmosphere. After 8 days of BrdU treatment cells were treated with various compounds (Azithromycin, Roxithromycin or Erythromycin) for another 3-5 days. BrdU or vehicle treatments were continued during the drug treatments as well.

### Sulphorhodamine B assay

After the incubation of the plates cell viability was measured by Sulphorhodamine B assay (SRB). The assay is based on the measurement of cellular protein contents. Cells were fixed with 10% Trichloroacetic acid (TCA) for 1 hour at 4^o^C, and were dried overnight at room temperature. Then, plates were incubated with SRB for 30 min, washed twice with 1% acetic acid and air dried for at least 1h. Finally, the protein-bound dye was dissolved in a 10 mM Tris, pH 8.8, solution and read using a plate reader at 540-nm.

### Autophagy and Cell cycle analysis

Autophagy (using Muse™Autophagy LC3-antibody based Kit, Merck Millipore) and cell cycle (Muse® Cell Cycle Kit, Merck Millipore) experiments were performed according to manufacturer`s instructions.

### Beta-Gal staining

Beta-Galactosidase staining of BrdU-treated MRC-5 cells was performed by Senescence β-Galactosidase Staining Kit (#9860, Cell Signaling Technology Inc.) and was done according to manufacturer`s protocol.

### Seahorse XFe96 metabolic flux analysis

Extracellular acidification rates (ECAR) and real-time oxygen consumption rates (OCR) for MCF7 cells were determined using the Seahorse Extracellular Flux (XF96) analyzer (Seahorse Bioscience, MA, USA) [30]. MRC-5 cells were maintained in MEM supplemented with 10% FBS (foetal bovine serum), 2 mM GlutaMAX, and 1% Pen- Strep. 40,000 cells per well were seeded into XF96-well cell culture plates, and incubated overnight at 37°C in a 5% CO2 humidified atmosphere. Next day, cells were treated with Azithromycin for 72 hours. Before the experiment, plate was washed with pre-warmed XF assay media (for OCR measurement, XF assay media was supplemented with 10mM glucose, 1mM Pyruvate and adjusted at pH 7.4). Cells were then maintained in 175 μL/well of XF assay media at 37°C, in a non-CO2 incubator for 1h. During incubation, 25 μL of of 80mM glucose, 9μM Oligomycin, 1M 2-deoxyglucose (for ECAR measure-ment) and 25 μL of 10μM Oligomycin, 9 μM FCCP, 10 μM Rotenone, 10 μM Antimycin A (for OCR measurement) in XF assay media was loaded into the injection ports of the XFe-96 sensor cartridge. During the experiment, the instrument injected these inhibitors into the wells at a given time point, while ECAR/OCR was measured continuously. ECAR and OCR mea-surements were normalized by protein content (Sulphorhodamine B assay). Data sets were analyzed by XFe-96 software, using one-way ANOVA and Student’s t-test calculations. All experiments were performed in triplicate.

### xCELLigence RTCA System (ACEA Biosciences Inc.)

Briefly, MRC-5 lung fibroblasts (vehicle alone and/or treated with 100 μM BrdU) were seeded in each well and employed to assess the efficacy of Azithromycin, using RTCA (real-time cell analysis), via the measurement of cell-induced electrical impedance. This approach allows the quantification of the onset and kinetics of the cellular response. Experiments were repeated several times independently, using quadruplicate samples for each condition.

### Statistical analyses

Statistical significance was determined using the Student’s t-test; values of less than 0.05 were considered significant. Data are shown as the mean ± SEM.

## Supplementary Material

Supplementary Table
