# Supplementary material for: Azithromycin and Roxithromycin define a new family of “senolytic” drugs that target senescent human fibroblasts
Source: Aging (Albany NY). 2018 Nov 14;10(11):3294–307. doi: 10.18632/aging.101633 (PMC6286845; doi:10.18632/aging.101633)
Supplement: Supplementary Table [file aging-10-101633-s001.pdf]

Supplemental Table S1

Ineffective compounds	Tested on	Concentration range used
Doxycycline	BJ	25 – 200 µM
Diphenyleneiodonium chloride	BJ	0.5 – 10 µM
Melatonin	BJ	200 – 400 µM
Aspartame	BJ	200 – 400 µM
Glucosamine	BJ	10 – 15 mM
Quercetin	BJ	10 – 100 µM
Dasatinib	BJ	0.1 – 1 µM
Chloroquine	BJ, MRC-5	100 – 200 µM
Erythromycin	BJ, MRC-5	100 – 200 µM
Clarithromycin	BJ, MRC-5	100 – 200 µM
Rapamycin	MRC-5	50 – 500 nM
Lycopene	MRC-5	25 – 50 µM
Alpha-lipoic acid	MRC-5	25 – 50 µM
